# Measles on the Edge: Coastal Heterogeneities and Infection Dynamics

**DOI:** 10.1371/journal.pone.0001941

**Published:** 2008-04-09

**Authors:** Nita Bharti, Yingcun Xia, Ottar N. Bjornstad, Bryan T. Grenfell

**Affiliations:** 1 Department of Biology, The Pennsylvania State University, University Park, Pennsylvania, United States of America; 2 Department of Statistics and Applied Probability, National University of Singapore, Singapore, Singapore; 3 Department of Entomology, The Pennsylvania State University, University Park, Pennsylvania, United States of America; 4 Fogarty International Center, National Institutes of Health, Bethesda, Maryland, United States of America; 5 Center for Infectious Disease Dynamics, The Pennsylvania State University, University Park, Pennsylvania, United States of America; University of Oxford, United Kingdom

## Abstract

Mathematical models can help elucidate the spatio-temporal dynamics of epidemics as well as the impact of control measures. The gravity model for directly transmitted diseases is currently one of the most parsimonious models for spatial epidemic spread. This model uses distance-weighted, population size-dependent coupling to estimate host movement and disease incidence in metapopulations. The model captures overall measles dynamics in terms of underlying human movement in pre-vaccination England and Wales (previously established). In spatial models, edges often present a special challenge. Therefore, to test the model's robustness, we analyzed gravity model incidence predictions for coastal cities in England and Wales. Results show that, although predictions are accurate for inland towns, they significantly underestimate coastal persistence. We examine incidence, outbreak seasonality, and public transportation records, to show that the model's inaccuracies stem from an underestimation of total contacts per individual along the coast. We rescue this predicted ‘edge effect’ by increasing coastal contacts to approximate the number of per capita inland contacts. These results illustrate the impact of ‘edge effects’ on epidemic metapopulations in general and illustrate directions for the refinement of spatiotemporal epidemic models.

## Introduction

The dynamic clockwork of measles epidemics is relatively well understood [1], [2]. Measles is a highly infectious virus and, in pre-vaccine developed countries, the strong acquired immunity following infection led to an average infection age of around 5 years [1], [3]. In both historic and current measles outbreaks, a brief primary infection followed by lifelong immunity results in violent epidemics. These epidemics extinguish themselves by depleting susceptible numbers, as hosts move irreversibly through susceptible-infected-recovered (SIR) classes. Subsequent epidemics start only when susceptible density has increased over time via births, resulting in repeated epidemic cycles interspersed with deep troughs of little or no measles incidence [4] (figure S1*b*). Seasonal patterns in aggregation and transmission can also force the timing and magnitude of these epidemic sequences [5], and this has been captured clearly in historical records of measles incidence for many countries [2].

Measles data from pre-vaccination England and Wales (1944–64) highlight spatial patterns in outbreaks and, therefore, also indirectly reveal patterns in human movement [6], [7]. A key driver of measles spatiotemporal dynamics is a threshold local population size, or critical community size (CCS), below which pre-vaccination measles will fail to persist and will go stochastically extinct (‘fade out’) between outbreaks. Epidemics in populations smaller than this threshold are subsequently reintroduced from other patches of a metapopulation. The CCS for measles in pre-vaccination England and Wales is estimated at approximately 300,000 individuals [8]. Outbreaks begin in cities that exceed the CCS, called ‘core’ cities and extend away from them, in traveling waves of infection [2], [6], [8]. These outward traveling waves identify core cities as epidemic drivers. The ‘coupling’ between patches quantifies the contact between them, which results in their degree of epidemic synchrony.

From previous studies, we understand that measles dynamics in London, as well as most of England and Wales, were driven by increased contact rates for children at the beginning of school terms [5]. These increases in contact rates led to higher transmission rates and ‘forced’ major biennial (every other year) epidemics. Susceptible density built up via births during non-epidemic years.

Despite complex geographic patterns of human habitation and movement (e.g. [9]), the simple gravity model appears to provide a fair approximation to many features of viral transmission. The gravity model parameterizes both the extent to which people move to large towns compared to small ones, and the spatial localization of their movements. These movements can occur for any reason and the model ignores geographic features such as desirability of each location as a vacation/weekend destination and the availability of public transportation. Moreover, it implicitly treats inland communities in a different fashion than coastal towns because neighboring communities surround inland towns whereas water partially surrounds coastal towns. To better understand the applicability and limitations of the gravity model, we were prompted by Savill *et al.* [10] to ask how well the model predicts **coastal** dynamics, as they point out that these are true ‘edges’ and therefore, are a unique case and a robust test of spatial models. We start with a simplified scenario: an artificial metapopulation with equidistant and equivalently sized inland and coastal towns arranged in a regular geometric shape. We use this simple system to analyze the model's predictions along the edges. Next, we study a more complex and realistic system using actual spatial, demographic, and epidemic data from England and Wales. We compared predicted and observed patterns of measles persistence in coastal locations to those of inland locations.

## Methods

### Local and Regional Scale Models

Within towns and cities (patches), hosts move from Susceptible (S) to Infected (I)to Recovered (R) classes irreversibly (full details and parameter values are given in [11]). We use a Time-series SIR model as follows:
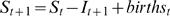
(1)

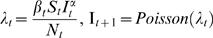
(2)Here, β_t_ is the seasonally varying transmission rate, which we have estimated from the data. The parameter α, when slightly less than unity (as here), is a conversion factor to go from continuous time to discrete time [12]. Each time step is two-weeks long to reflect the average infectious period of the measles virus. Cases enter the infected class at the beginning of a time step. Two weeks later, they exit the infected class and enter the recovered class in the next time step. We define a fadeout as one biweek (a two-week interval) with zero cases reported in a location. In any one location, the absence of a fadeout signifies a possible unbroken local chain of transmission and we assume measles has persisted locally. Conversely, cases following a fadeout, or local extinction, indicate that the virus was locally reintroduced.

Reintroductions occur when the movement of individuals results in transmission events between patches. In order to quantify this movement, we calculate patch connectivity using a gravity model [2], [7]. Specifically, we represent the expected movement of individuals from patch *j* to patch *i* as:
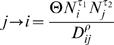
(3)Here, N*_i_* and N*_j_* are the population sizes of towns *i* and *j*, respectively, D_ij_ is the Euclidean distance between towns *i* and *j*, ρ is a power describing how flux decays with distance, τ_1_ and τ_2_ are immigration and emigration exponents, respectively, and Θ is a coefficient of spatial coupling. Movement between patches is most likely to occur over short distances and towards patches with large populations. Xia *et al.* [7] used a spatially explicit metapopulation model for England and Wales with gravity coupling to estimate the coefficients as ρ = 1, τ_1_ = 1, τ_2_ = 1.5, and Θ = 4.54×10^−9^ km/person^1.5^/biweek.

Savill *et al.* [10] examined the 2001 UK foot and mouth disease epidemic and determined that Euclidean distance was a better predictor for transmission risk than shortest or quickest route. While transportation of infectious materials likely occurred over roads in the foot and mouth epidemic, multiple transmission routes over long distances made Euclidean distance a significantly better predictor of simple spatial transmission risk. For these reasons, we use Euclidean distance here.

This gravity framework successfully captures the overall defining features of pre-vaccination measles dynamics in England and Wales, particularly the relationship between measles persistence and spatial synchrony with local population size and isolation [7]. Disease models with similar distance-weighted size-dependent coupling have recently been used to capture spatiotemporal epidemic spread successfully in a number of other systems [10], [13], [14], [15], [16].

### Simple Case: Artificial Metapopulation Model

**Figure 1:**
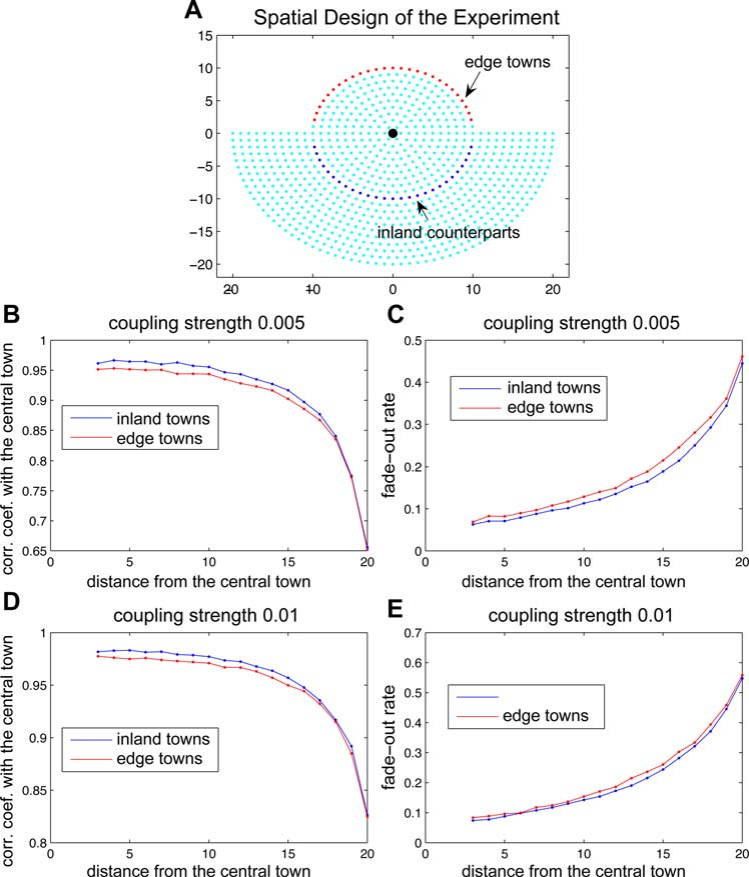
Simulated metapopulation for edge analysis. *(a)* The spatial distribution of towns (all dots), the large black dot represents a core city or central town (analogous to London) and the edge towns in red (analogous to coastal towns) have inland towns in navy (analogous to any non-edge towns) that are equidistant from the central town and similar in size. *(b)*, *(c)* The model always predicts a reduced correlation coefficient and an increased fadeout rate between an edge town and the central town than between a similar inland town and the central town. The bias is significant, although it looks slight here, and is even stronger in the real model predictions for England and Wales (figure 2*b* and 3*b*) *(d)*, *(e)* This can be corrected by increasing the coupling strength (Θ in equation 3).

We created a metapopulation with one core city (a city which exceeds the CCS) surrounded by many cities below the CCS (figure 1*a*). Our intentions with this simple artificial population were to isolate and identify the effects of being an edge city by holding population size and distance to core city constant and equal between edge and non-edge patches. Therefore, this metapopulation included 27 cities along the edge of the system that were all equal in size and equidistant from the core city. The metapopulation also included an equivalent set of 27 non-edge cities, or inland patches, also equal in size and equidistant from the core city. We introduced a simulated measles epidemic into this artificial metapopulation. We ran the simulation for 5000 biweeks and used the predictions from the final 4000 biweeks. We chose an R_0_ value of ∼30, invariant of community size ([11] their figure 8 and p. 180) and an alpha slightly less than unity. We gradually increased the coupling strength from .005 to .02 (the fitted value for this parameter falls within this range) and used seasonal term time forcing, as observed in the data. We compared the model predictions for disease persistence and outbreak correlation to the core city between these edge and inland cities from our artificial metapopulation.

### Actual Case: England and Wales Metapopulation

**Figure 2:**
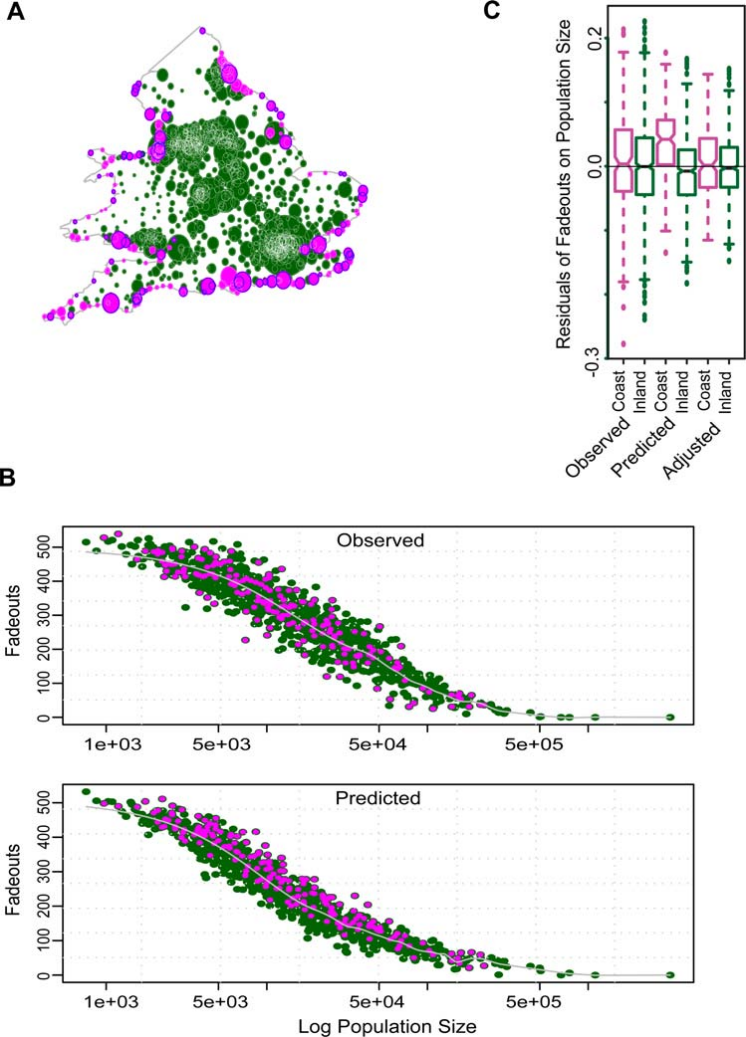
The model predicts more coastal fadeouts than observed for England and Wales. *(a)* Map of England and Wales showing the location of each of the 952 towns included in this study. Green circles with white outlines are inland towns, pink circles are coastal towns. Area of each dot is correlated to population size. *(b)* Total number of fadeouts against population size as observed (top) and as predicted by gravity model (bottom). In the data, the coastal fadeouts (pink) are distributed among the inland fadeouts (green). In the model predictions, the coastal fadeouts are clustered near the top of the inland distribution. *(c)* Boxplots showing coastal (pink) and inland (green) pairs of residuals of fadeouts on population size. Left: observed; center: gravity model predictions; right: gravity model predictions with spatial coupling increased for the entire coast.

Our data consist of biweekly cases, population size, and birth rate from each of 952 towns in England and Wales (figure 2*a*) from 1944 to 1964. We used latitude and longitude coordinates to determine the Euclidean distance between each pair. We defined coastal towns as those within 5 km of the edge of the island and we identified 184 of these.

To investigate edge effects in England and Wales, we first fit the gravity model (equation 3) to fortnightly data for measles from 1944 to 1964 for all 952 towns in England and Wales. The gravity model parameters (Θ, τ_1_, τ_2_, and ρ) were estimated by a combination of short and long term predictions, as described by Xia *et al.* [7] and discussed above. (For a more detailed description of how the gravity model was fit, please see [7].) We ran gravity model simulations for twenty-one consecutive years with each year consisting of 26 biweeks in order to test the null hypothesis that the simulated persistence and dynamics of coastal towns and non-coastal towns of the same size would be similar.

### Seasonality

We considered the possibility that coastal cities may reach peak densities (and therefore have elevated contact rates) during the summer months, when travelers are preferentially drawn to the coast [17]. To investigate the impact of specific coastal seasonality on measles epidemics, we compared coastal outbreak seasonality to inland outbreak seasonality from the data. We compared the average timing and duration of actual epidemic peaks and troughs for the entire 21-year period.

### Public Transportation

While there are no specific records of human movement to and from the coastline during the time period studied, we used annual public transportation passenger volume from that time in England and Wales [18]. As a proxy for contact rates, we analyzed these data for interesting patterns or anomalies, with respect to each coastal town's population size and location. We were unable to obtain bus passenger volume, road use data or any seasonal movement data, which would have complemented annual train passenger data to more completely show movement patterns.

## Results

### Simple Case: Artificial Metapopulation Model

The gravity formulation uses distance and population size to capture complex patterns of human movement in a relatively crude way. These movements are important because they drive epidemic spread between patches of metapopulations. To understand how the model specifically treats edges of metapopulations, we used an artificial metapopulation with ‘edge’ and ‘non-edge’ towns that were similar in size (all were below the CCS) and in equidistant from the core city (figure 1*a*). It is important to note that each coastal town we identified in the data was below the CCS and therefore too small to sustain a continuous chain of measles transmission in the absence of re-introductions. We introduced a simulated measles epidemic into our artificial metapopulation and focused on the model's predictions of persistence in ‘edge’ towns and the equivalent ‘non-edge’ towns.

In our artificial metapopulation, the model predicts that edge town epidemics stochastically fade out more frequently than inland town epidemics. The edge towns have fewer contacts and re-introductions than inland towns due to the predicted movement of individuals within a gravity model framework. Reduced contacts would lead to a difference in fadeout frequency between edge and inland towns. These edge town epidemics are also less correlated with the core city than are the inland town epidemics, another likely result of reduced contacts (figure 1*b*). Figure 1 illustrates this ‘edge effect’ by using our simple model core-satellite disease metapopulation [7] to show that locations along the edge of the system were predicted to experience a smaller flux of infective sparks than their inland counterparts. We are confident that the increased fadeouts and reduced correlation along the edge were due to reduced contacts because solely increasing the per capita contacts for towns along the edge is sufficient to result in comparable fadeouts between edge and inland equivalents (figure 1*c*, 1*e*) and to increase edge epidemic correlation with the central core town (figure 1*b*, 1*d*).

### Actual Case: England and Wales Metapopulation

England and Wales provide an excellent opportunity to explore how the gravity model applies to measles and real coastal towns, or ‘edges.’ The ocean surrounding Great Britain was a relatively impenetrable geographic barrier that was unlikely to introduce many measles cases, relative to the impact of child movement inland. This allows us to observe a fairly epidemically autonomous system to analyze the true edge effects of human movement in a metapopulation and epidemics on an island (figure 2*a*).

The gravity model predicted a significantly greater difference in fadeouts for coastal towns in England and Wales than for inland towns (corrected for population size) than we observed in the data (figure 2*b* and 2*c*, figure S1). These model predictions show a greater bias than was seen in both our simulated metapopulation and simple coastal system models. Importantly, model predictions showed no spatial bias or geographic clustering of overestimated fadeouts (figure S2). *This indicates that the basic gravity metapopulation is unable to capture the relatively high level of stochastic persistence seen in coastal measles. We now explore possible extrinsic and mechanistic epidemiological explanations for this discrepancy*.

### Seasonality

**Figure 3:**
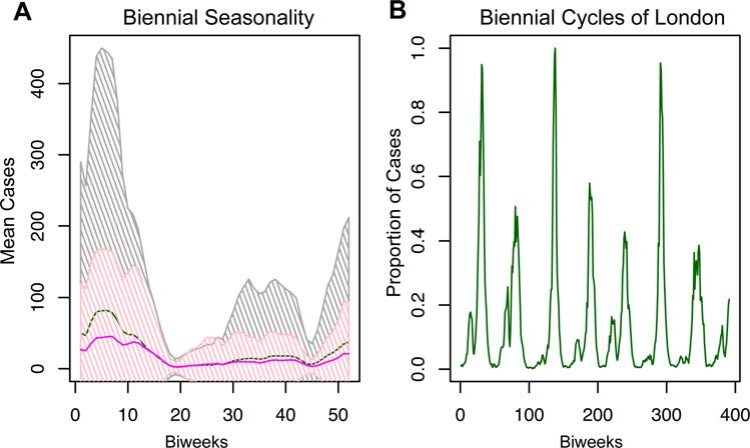
Epidemic Seasonality: Inland and Coastal. *(a)* The biennial epidemic seasonality does not differ between the inland towns (dark green line indicates inland seasonal mean, light gray shading is +/− one standard deviation) and the coast (magenta line indicates coastal seasonal mean and pink shading is +/− one standard deviation); both peak at the same times and the cycle repeats every two years. Time is shown as biweeks on the x-axis, where 26 biweeks are equal to one year. *(b)* The biennial outbreaks in London, the largest city in this system, drive the cycles of England and Wales. Susceptibles are depleted during large outbreaks and accumulate during epidemic troughs until the next outbreak. Major peaks occur at the beginning of the school term every second year. Time is shown as biweeks on the x-axis, where 26 biweeks are equal to one year.

The epidemic seasonality of coastal and inland towns were not significantly different; the epidemics both began and peaked at similar times during the same years, which correspond to school terms (figure 3*a*). The similar timing of epidemics implies that contact rates between inland and coastal locations were high enough to cause inland core cities to spark coastal epidemics. Extremely low contact rates between inland and coastal towns would have resulted in highly uncorrelated measles epidemics along the coast of England and Wales, relative to inland town epidemics. The data show that these contact rates were higher than predicted by the gravity model because the inland and coast were in epidemic synchrony.

### Public Transportation

**Figure 4:**
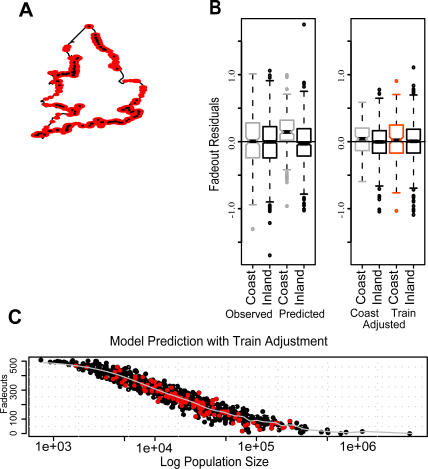
Adjustments to gravity model using train use data. *(a)* Map of England and Wales. Red dots show coastal locations, size of dots reflects amount of train use, black dots in the center of red dots indicate train use >10,000 weekly passengers. *(b)* Left Panel: Left shows data, right shows initial gravity model predictions, gray = coast, black = inland. Right Panel: Left shows model predictions with increased spatial coupling along entire coast, right shows model with increased spatial coupling for towns along coast with >10,000 weekly train passengers (red), gray = coast, black = inland. *(c)* Total number of fadeouts against log population size as predicted by gravity model when the spatial coupling coefficient is increased for only the coastal towns which average >10,000 weekly train passengers per week. This adjustment results in an even distribution of coastal fadeouts (red), as they appear in the data. Initial model predictions show coastal fadeouts are clustered along the top of the distribution (figure 2*b*).

These data revealed no abnormal relationship between train use and measles persistence that could not be explained by population size, a component that the gravity model already considers (figure S3 and figure 4). We also saw no explanatory spatial patterns in the passenger train volume.

### Model Adjustments

Although we found no statistically significant difference between train use and population size (figure S3*b* and S3*c*), we adjusted the spatial coupling coefficient for only the coastal towns with passenger train volume that exceeded 10,000 passengers per week (which we categorized as high train use) and repeated the simulations. We used least squares to compare our adjusted model predictions to the data for the 109 coastal cities with high train use that we identified (figure S3*a* and figure 4*a*). We found that increasing the basic coupling parameter (Θ) by 1.3 times the initial fitted value allowed the model to more accurately predict the fadeouts along the coast. The model simulations with this adjustment did not significantly overestimate the total number of coastal fadeouts (figure 4*b* and 4*c*).

The volume of passenger traffic clearly highlights the mobility of individuals in England and Wales at this time. In order to model this level of host mobility, we repeated the model simulations with another adjustment. We optimized the gravity model to reflect equal numbers of individual contacts in coastal and inland towns. The data suggested a conservation of contacts created a more accurate map of social space over geographic space. We addressed this by minimizing the difference of the sum of squares between adjusted model predictions and the data. For all 184 coastal towns, we found the simulations fit best when we increased the spatial coupling by 1.3 times our initial Θ for all 184 coastal towns. With this adjustment, the model simulations no longer showed a bias towards coastal fadeouts and predictions more accurately matched the observed fadeout rate distribution (figure 2*c* and figure S1).

## Discussion

In this study, we demonstrate a discrepancy between modeled and observed coastal epidemics and we ask what drives this breakdown of the gravity model assumptions. Because our question address spatial transmission, detailed human movement data to parallel the epidemic time series would be ideal for comparison but these are not available, particularly for children, during the 1940s and 50s [3]. Instead, we used local population heterogeneities to test the gravity model predictions of epidemic persistence and synchrony.

Our initial, basic gravity model may have incorrectly predicted coastal persistence for two possible reasons. First, the towns along the coast may have had contact rates that were different from those of inland towns. For example, we considered the possibility that coastal locations may have experienced relative isolation-by-distance and low contact rates for most of the year, alternating with high contact rates during the summer months, due to travelers [17]. In inland towns, contact rates are highest at the beginning of each school term, when epidemics take place. Cyclic demographic flux could cause the model to underestimate seasonal movement and coastal contacts, resulting in overestimated fadeout rates caused by not considering summer cases and predicting only school term epidemics, sparked by core cities. In this situation, coastal towns would show measles outbreaks in the summer, unique from the rest of the island, where contact rates and epidemics rise and peak during the school term. The data do not show this.

If coastal towns showed epidemic seasonality that indicated summer outbreaks, this would imply that they were somewhat isolated from inland towns and were not influenced by inland epidemic cycles as a result of unique contact rates. However, our analysis shows that coastal towns measles epidemics followed the term time forcing of the large inland cities and that coastal towns were not at all isolated from inland towns. Therefore, the data show us that coastal epidemic cycles were likely driven by core cities, which were only found inland, indicating that coastal and inland towns did not have different seasonal contact rates (figure 3*a*). It has further been shown that the coast was an attractive location for suburban residences year round, as well as for seasonal holidays [19], not a continuously isolated edge as the model predicted in both our simulated ‘artificial metapopulation model’ and our England and Wales simulations. The data clearly show that coastal towns did not have reduced contact rates with inland towns.

A second possible reason that the gravity model overestimated fadeouts along the coast is that coastal and inland per capita contact rates are relatively similar. The distance-weighted, size-dependent spatial coupling element of the basic gravity model will always predict lower overall contact rates for coastal than for inland towns. Because coastal towns are partially surrounded by water, they have fewer populations at close proximity (small *D_ij_* in equation 3), which greatly impacts the flux of infection between towns. However, if each coastal town approximately averages the same number of contacts per capita as inland locations (as the public transportation data suggest in figure S3), and the model is unable to map social space over geographic space by assuming the opposite, then the prediction of reduced contacts along the coast would create a false “edge effect” of increased fadeouts. It is both unrealistic and counterintuitive to assume reduced individual coastal contacts; living along the coast does not reduce the need, for example, for medical attention, commerce, or social companionship. If observed contact rates are reasonably similar between coastal and inland towns, the model will underestimate contacts, transmission, and persistence along the coast. In this case, the observed coastal epidemic seasonality would not differ from inland seasonality, as it does not in this system.

If host mobility resulted in high contact rates along the coast year-round, even for distant cities, this would result in multiple measles introductions during local epidemics troughs. While these introductions would not have sparked new measles epidemics because of low susceptible density resulting from regular biennial outbreaks, they would have sparked isolated cases and led to decreased fadeout rates in coastal towns. However, it is very difficult to determine actual contact rates; even though we were able to obtain passenger train use volume, we did not have bus or road use data. Further, even with all those data, we would still fail to quantify the actual movement of children. Thus, while our train use data give us a good idea of host mobility and train use by town size, it is still only a vague approximation of the contacts we are actually interested in.

In the gravity model, the spatial coupling coefficient (Θ, equation 3) represents the amount of human movement from one town to another; as Θ increases, contacts increase and spatial synchrony increases. Based on our model predictions, the spatial coupling parameter estimation fits inland towns well but underestimates the connectivity of coastal towns.

In figure 4*b*, we compare the residuals of the fadeouts on population size between the observed data, initial gravity model predictions, adjusted gravity model predictions for high train use coastal towns, and adjusted gravity model predictions for all coastal towns. Although the high train use adjustment gravity model predicts a slight bias towards coastal fadeouts, it corrects for most of the bias in the initial, unadjusted gravity model predictions and more accurately reflects the observed data. When we increased the coastal spatial coupling coefficient to more accurately map social space over geographic space for the purpose of increasing coastal contact rates, the adjustment corrected for the model's bias of reduced coastal contacts and increased coastal fadeouts (figure 1*d* and 1*e*, figure 2*c*, and figure 3).

### Conclusions

Contact from core cities to coastal regions introduced isolated measles cases during the troughs between epidemics. These stochastic introductions did not lead to out-of-phase epidemics along the coast; instead they resulted in a low level of persistence [20]. When this occurred, coastal towns did not fade out as the model predicted because of the model's inaccurate assumption that locations at the edge of a system have reduced contact rates, simply because of their position. The observed data do not support this assumption, implying that (at least childhood) behavior and movement in this landscape do not isolate geographic edges.

The adjustments we have shown here crudely illustrate the gravity model's potential to accommodate spatial heterogeneities and host behavior in stochastic metapopulations by identifying important geographic features, which can influence host mixing behavior and affect disease transmission [21]. The spatial coupling coefficient for edges can be increased when host mobility results in reduced isolation-by-distance.

In realistic landscapes, habitats often include variation in accessibility, land quality and resource availability. Populations establish centers and edges with respect to these features. The methods presented here can be applied as a first step to understanding disease dynamics and host movement across heterogeneous landscape peripheries. Dissecting the applied implications of these results is an important area for future work, especially in developing countries [22]. It is clear that more sophisticated methods need to be developed to address these specific issues with spatial models but these findings make a satisfactory first step in identifying the problem and exploring solutions.

## Supporting Information

Figure S1 Residuals from ‘proportion of fadeouts against log population size’ against log population size. Another view of the observed data, the bias in the original model predictions, and the model predictions with our adjustments.
(0.26 MB DOC)

Figure S2 Spatial distribution of coastal fadeouts from model predictions. The model is not spatially biased in predicting fadeouts. Fadeouts were overestimated as well as underestimated along all parts of the coast and in all population sizes.
(0.13 MB DOC)

Figure S3 Measles persistence, population size, and train use along the coast. Population size, measles persistence, and train use are all strongly correlated for coastal towns. This is true in both the observed data and model predictions. There was no unsual movement of people (as approximated by train use) that affected coastal measles persistence in a way that could not be explained by population size.
(0.18 MB DOC)
