# Supplementary material for: Measles on the Edge: Coastal Heterogeneities and Infection Dynamics
Source: PLoS One. 2008 Apr 9;3(4):e1941. doi: 10.1371/journal.pone.0001941 (PMC2275791; doi:10.1371/journal.pone.0001941)
Supplement: Figure S1 — Residuals from ‘proportion of fadeouts against log population size’ against log population size. Another view of the observed data, the bias in the original model predictions, and the model predictions with our adjustments. (0.26 MB DOC) [file pone.0001941.s001.doc]

Measles on the Edge: Coastal Heterogeneities and Infection Dynamics

Supporting Information File #1

Residuals from ‘proportion of fadeouts against log population size’ against log population size


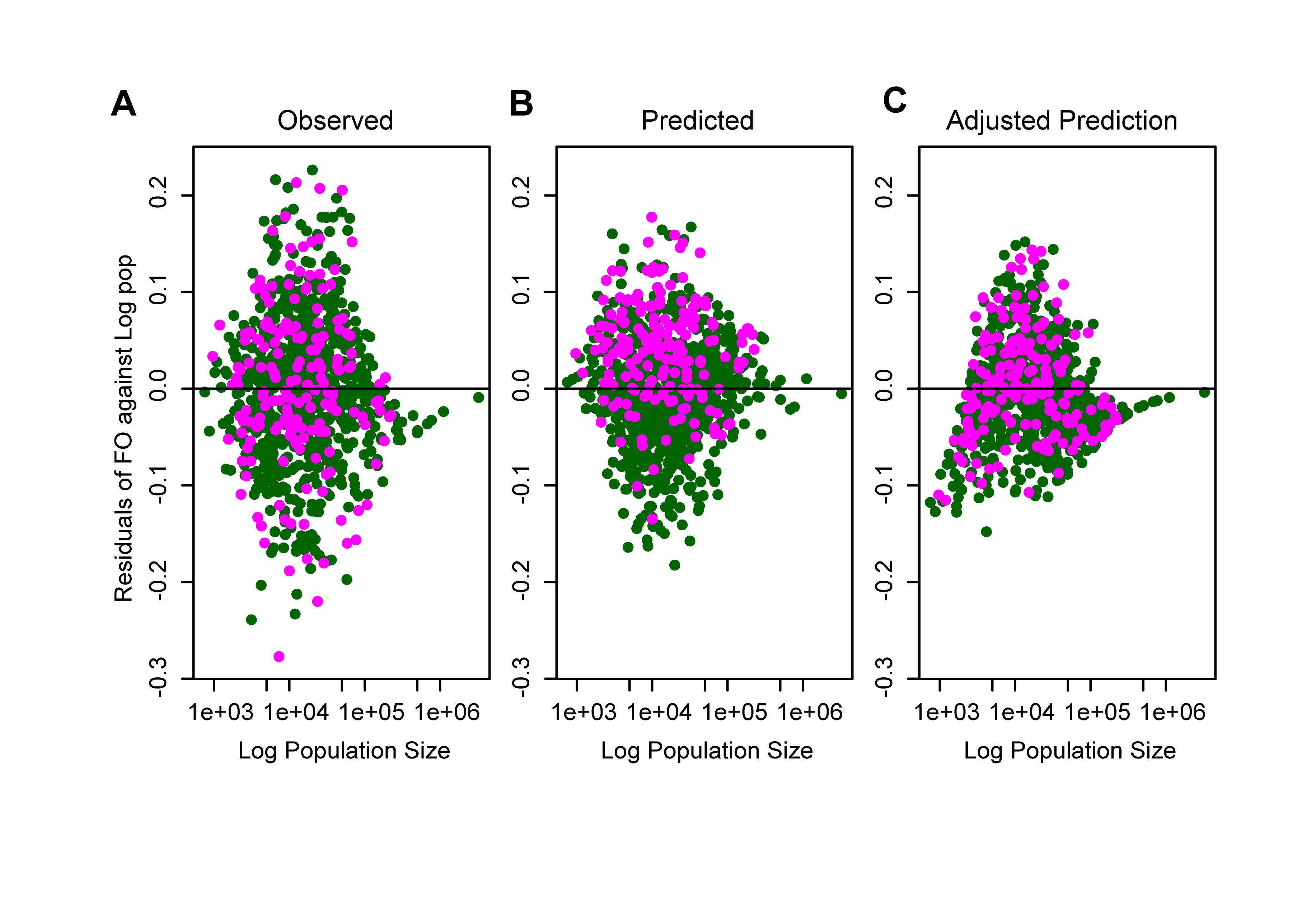


Figure S1 Residuals from ‘proportion of fadeouts against log population size’ against log population size

The center panel *(b)* shows that the gravity model predicts a strong bias for coastal towns of all population sizes to fadeout more than the data show *(a)*. The panel on the right *(c)* shows that our model adjustment corrects for this bias and the distribution of predicted coastal fadeouts is similar to that of inland towns. Inland in dark green, coast in magenta.
