# Supplementary material for: Measles on the Edge: Coastal Heterogeneities and Infection Dynamics
Source: PLoS One. 2008 Apr 9;3(4):e1941. doi: 10.1371/journal.pone.0001941 (PMC2275791; doi:10.1371/journal.pone.0001941)
Supplement: Figure S2 — Spatial distribution of coastal fadeouts from model predictions. The model is not spatially biased in predicting fadeouts. Fadeouts were overestimated as well as underestimated along all parts of the coast and in all population sizes. (0.13 MB DOC) [file pone.0001941.s002.doc]

Measles on the Edge: Coastal Heterogeneities and Infection Dynamics

Supporting Information File #2

Spatial distribution of coastal fadeouts from model predictions


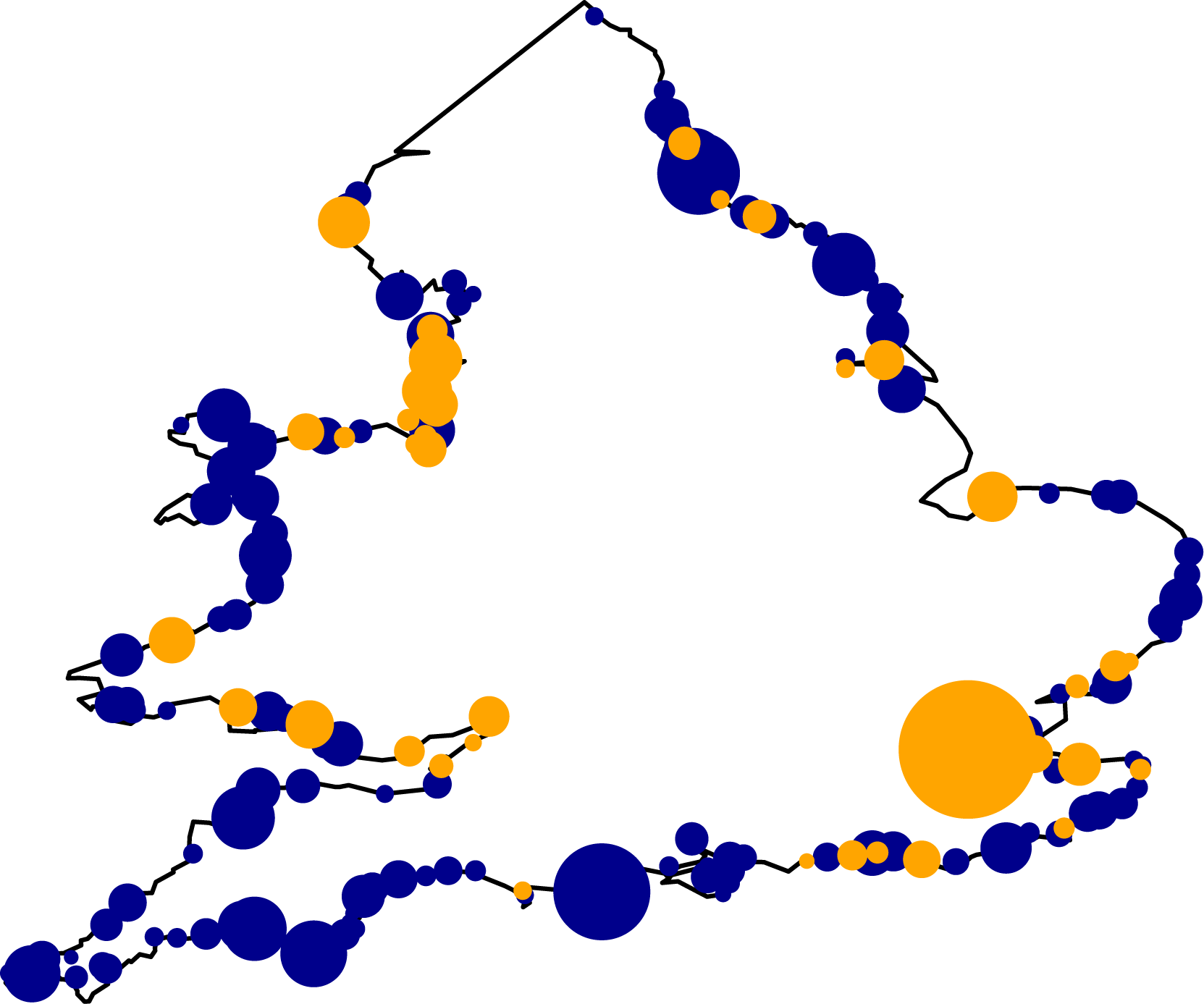


Figure S2 Spatial distribution of coastal fadeouts from model predictions

Navy dots show coastal towns that the model predicts will fade out more than the average number of predicted fadeouts. Yellow dots show the towns that are predicted to fade out less than the average number of predicted fadeouts. The navy dots are found along all sections of the coast and do not reveal any spatial pattern. Similarly, the yellow dots show no spatial clustering. This shows that the model is not spatially biased in its predictions. The size of each dot is proportional to population size.
