# Supplementary material for: Measles on the Edge: Coastal Heterogeneities and Infection Dynamics
Source: PLoS One. 2008 Apr 9;3(4):e1941. doi: 10.1371/journal.pone.0001941 (PMC2275791; doi:10.1371/journal.pone.0001941)
Supplement: Figure S3 — Measles persistence, population size, and train use along the coast. Population size, measles persistence, and train use are all strongly correlated for coastal towns. This is true in both the observed data and model predictions. There was no unsual movement of people (as approximated by train use) that affected coastal measles persistence in a way that could not be explained by population size. (0.18 MB DOC) [file pone.0001941.s003.doc]

Measles on the Edge: Coastal Heterogeneities and Infection Dynamics

Supporting Information File #3

Measles persistence, population size, and train use along the coast


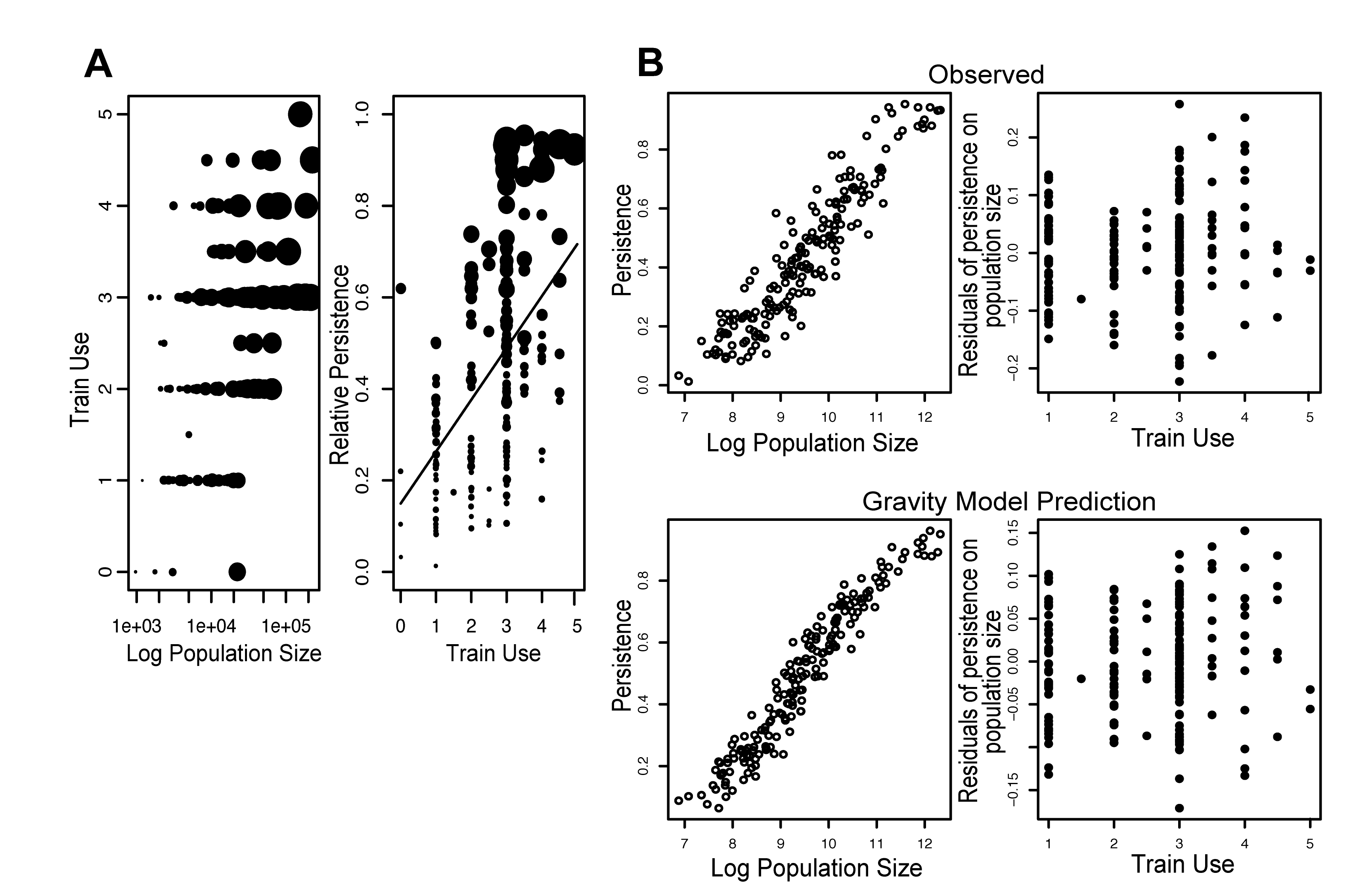


Figure S3 Measles persistence, population size, and train use along the coast

*(a)* Left: Train use against log population size for each coastal town. Size of dot reflects relative persistence. Right: Relative Persistence against train use, size of dots reflect population size.

*(b)* The top two panels show the data. Left: Log of population size is strongly correlated with persistence. Right: The effect of train use on the residuals of persistence on population size is not significant (P = .08279). The bottom two panels show the gravity model predictions. Left: Log of population size is strongly correlated with persistence. Right: The effect of train use on the residuals of persistence on population size is not significant (P = .08191).
